# The Effect of Disinfection by Spray Atomization on Dimensional Accuracy of Condensation Silicone Impressions

**DOI:** 10.5681/joddd.2010.031

**Published:** 2010-12-21

**Authors:** Fariba Saleh Saber, Nader Abolfazli, Maryam Kohsoltani

**Affiliations:** ^1^ Assistant Professor, Department of Prosthodontics, Faculty of Dentistry, Tabriz University of Medical Science, Tabriz, Iran; ^2^ Associate Professor, Department of Periodontics, Faculty of Dentistry, Tabriz University of Medical Science, Tabriz, Iran; ^3^ Post-graduate Student, Department of Oral & Maxillofacial Pathology, Faculty of Dentistry, Tabriz University of Medical Science, Tabriz, Iran

**Keywords:** Condensation silicone, dimensional accuracy, disinfection, elastomer, impression

## Abstract

**Background and aims:**

The condensation silicone impression materials are available, but there is little knowledge of their accuracy after disinfection. The objective of this study was to evaluate the effect of the disinfection by spray atomization on dimensional accuracy of condensation silicone impressions.

**Materials and methods:**

Impressions were made on a stainless steel master model containing a simulated two complete crown preparation with an edentulous space interposed using Spidex® and Rapid® impression materials. 44 impressions were made with each material, of which 16 were disinfected with 5.25% sodium hypochlorite, 16 were disinfected with 10% iodophor and 12 were not disinfected. Three dimensional measurements of working casts, including interpreparation distance, height, and diameter, were calculated using a measuring microscope graduated at 0.001 mm. Dimensional changes (mm) between the disinfected and non-disinfected working casts were compared. One-way analysis of variance (ANOVA) was employed to analyze the data (α=0.05).

**Results:**

Disinfection of each condensation silicone material by spraying atomization with two different disinfectant material resulted in significant change in interpreparation distance (p<0.05). Changes in height and diameter were only significant in Spidex® impressions (p<0.05).

**Conclusion:**

Significant changes in the mean dimensions were seen as a result of disinfection by spraying; however, the dimensional changes do not seem great enough to cause critical positional distortion of teeth when fixed partial denture restorations are made.

## Introduction

Impression materials are used to register or reproduce the form and relation of the teeth and the surrounding oral tissues.^1^ Elastomeric impression materials set by either condensation or addition polymerization reactions.^2^ In prosthodontics, impression material and prosthesis that have been exposed to infected saliva and blood pose a main source of cross-contamination and additional problems in controlling cross-infection between dental office and laboratories.^3,4^ In view of the infectious carrier state of a significant proportion of the population and current trends in cross-infection control, disinfection of the impressions is seriously recommended by the American Dental Association (ADA) and the Centers for Disease Control to prevent possible transmission of infectious diseases.^5,6^Despite the necessity of additional control procedures and disinfection during making and handling of dental impressions immediately after removal, it should be ensured that such procedures do not alter dental impressions. To issue guidelines regarding impression disinfection, the ADA determined the antimicrobial agents to be used for different impression materials and the time, dilution, and temperature needed for the optimal performance of each agent.^6^ The disinfecting process should be proper, but should not have an adverse effect on the dimensional stability or the surface detail of the impression.^7^ The effects of disinfection methods on the accuracy of different impression materials have been investigated.^8-18^ Regardless of different methodologies used, previous studies have also shown that the immersion disinfectant has no clinically relevant effect even on hydrophilic materials;^19-21^,however, other studies have indicated that the dimensional stability of hydrophilic materials is adversely affected by immersion.^9,19^ Other studies have also evaluated the possible damage to the quality of the elastomeric materials impression according to the disinfecting products, methods, and time used.^22-25^,

However, there is limited literature on the effect of disinfection by spraying on the dimensional accuracy of the condensation silicon impression materials. Although condensation silicon impression material is inherently unstable chemical structure because of evaporation of volatile by-products from condensation reaction, it is commonly used in Iran. The purpose of this study was to evaluate the effect of the disinfection by spray atomization on dimensional accuracy of two currently available, commonly used condensation reaction silicon impression materials in Iran. The null hypothesis was that there would be no differences in the accuracy of working casts made with the disinfected and non-disinfected impressions.

## Materials and Methods

**Figure 1:**
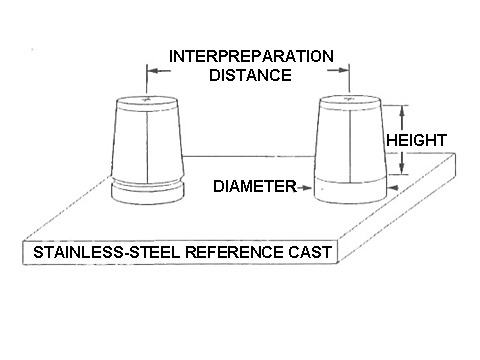
Schematic representation of stainless steel master cast representing preparations for fixed partial denture. Measurements of changes in dimension were made at locations labeled interpreparation distance, height, and diameter.

A stainless steel master cast which was used to provide a dental replica of two teeth prepared for complete crowns with an edentulous space interposed, represented the clinical situation of two abutment teeth prepared to receive crown retainers splinted to two pontic forms. The effect of the two different disinfectants on the surface of the condensation reaction silicon impressions by spray atomization was evaluated measuring three clinically dimensions on dental stone casts recovered from the impressions of the standard master cast. The experimental methods have been previously described and a schematic representation of the standard master cast is illustrated in Figure 1.^26,27^

The impression materials evaluated were two condensation reaction silicon impression materials (Spidex®, Coltene AG, Altstatten, Switzerland; Rapid®, Coltene AG, Altstatten, Switzerland) used with the putty-wash technique. Perforated metal stock trays with retentive rims (Omnident GmbH, Rodgau, Germany) were used to make all impressions by a single investigator. All materials were mixed at room temperature (25°C) and placed within the working time recommended by the manufacturer. The impressions were allowed to polymerize approximately three times longer (15 minutes) than the time recommended by the manufacturer to ensure adequate polymerization occurred at room temperature.^26^ A total of 44 impressions were made with each material. 16 impressions were disinfected with one disinfectant and another 16 impressions were disinfected with a second disinfectant. 12 impressions with each material were used as non-disinfected controls. 10% iodophor (Biotrol Inc., North Salt lake ,Utah) and 0.525% sodium hypochlorite (Lacroix, Colgate-Palmolive, France) solutions were used for disinfection by spray atomization. After the impressions were removed from the master model, they were rinsed for 10 seconds under running water, and then air dried. The impressions were sprayed by the disinfectant and stored in a plastic sealed bag for 10 minutes, then rinsed again for 10 seconds under running water, air dried, and left for an additional 110 minutes before impression pouring.^11^ The non-disinfected impressions were left for 120 minutes before pouring gypsum casts. Type IV gypsum (Towerock, Kettenbach GmbH, Germany) was used to make the working casts. The recommended ratio of 20 ml of distilled water to 100 g of powder was used. The powder and water was first mixed by hand for 10 seconds, then vacuum mixed (Multivac 4; Degussa GmbH, Hanau, Germany) for an additional 30 seconds. The gypsum was vibrated into the impressions and allowed to set for 60 minutes. The casts were left at room temperature for 24 hours to dry after being removed from the impressions.

Measurements of three clinical dimensions including interpreparation distance, height, and diameter were a made by one calibrated examiner using a measuring microscope (Measurescope MM- 400; Nikon GmbH, Düsseldorf, Germany) capable of measuring to 1 μm. Each measurement was repeated three times on each stone cast, and the mean of these measurements was recorded.^26,27^ The measurements were made blinded to the type of impression material and to the disinfection condition. The degree of dimensional distortion observed in the stone replicas was expressed as a percentage of change from the measurement values for the standard.

For an alpha level of 0.05, sample size for each group was calculated to achieve a power of 95% and efficient size of 0.5%. The data collected from the investigation of two impression materials treated with two disinfectants, were analyzed for each clinical dimension with an analysis of variance (ANOVA) by using SPSS 16.0 statistical software (SPSS, Inc, Chicago, Ill, USA). Significance level was set at α=0.05.

## Results

Table 1 shows the mean values and standard deviations of the dimensional changes of each impression material with different disinfectant agents. The comparative results of each disinfected impression material with similar non-disinfected material are presented in Table 2. There were no statistically significant differences in height and diameter between disinfected and non-disinfected Rapid® impression material, although the difference in interpreparation distance was significant. In Spidex® group, disinfection of impressions resulted in significant changes in interpreparation distance and diameter. There was also a significant difference in height between non-disinfected and iodophor-disinfected Spidex® groups.

**Table 1:** Means and standard deviations of height (mm), interpreparation distance (mm), and diameter (mm) according to the disinfectant solution and impression material

Material/Group	Height (mm)	Interpreparation distance (mm)	Diameter (mm)
Rapid/Control	8.68 ± 0.02	40.43 ± 0.08	9.64 ± 0.04
Spidex/Control	8.69 ± 0.01	40.46 ± 0.08	9.58 ± 0.05
Rapid/Iodophor	8.67 ± 0.03	40.52 ± 0.09	9.62 ± 0.03
Spidex/Iodophor	8.66 ± 0.02	40.55 ± 0.04	9.66 ± 0.03
Rapid/Hypochlorite	8.67 ± 0.01	40.60 ± 0.05	9.62 ± 0.03
Spidex/Hypochlorite	8.67 ± 0.01	40.58 ± 0.04	9.63 ± 0.02

**Table 2:** Comparison of dimensional changes of working casts relative to the control groups in the three dimensions evaluated

Measurements	Rapid/Iodophor	Rapid/Hypochlorite	Spidex/Iodophor	Spidex/Hypochlorite
Rapid/Control				
Height	P=0.99	P=0.98	—	—
Diameter	P=0.53	P=0.56	—	—
Interpreparation distance	P=0.01^ * ^	P<0.0005^ * ^	—	—
Spidex/Control				
Height	—	—	P=0.04^ * ^	P=0.30
Diameter	—	—	P<0.005^ * ^	P=0.30^ * ^
Interpreparation distance	—	—	P=0.01^*^	P=0.00^*^

*: Statistically significant.

In spite of significant differences between samples with various disinfection protocols, it is important to note that all the dimensional accuracy measurements were lower than 0.4%, within the ADA specification 19 requirements (≤0.5% dimensional change).

## Discussion

The null hypothesis was that there would be no differences in the accuracy of working casts of the two impression materials after disinfection by spraying. This hypothesis was rejected for the Spidex® impressions, since there were statistically significant differences among disinfected and non-disinfected dental stone casts, but was partially accepted for the Rapid® impressions. In most situations, the detected differences were small in magnitude and of minor clinical significance, considering other factors such as tooth mobility,^28^ mandibular deformations during opening,^29^ potential inaccuracies during laboratory processes,^30,31^ and the clinically accepted values for marginal gaps of crowns (150-100 µm).^32,33^

In this study, spray technique was used for disinfection of the impressions. The spray technique has shown similar antimicrobial activity compared to the immersion method;^17^ however, unlike the immersion method, it does not cause dimensional changes.^34^ According to the ADA specifications for elastomeric impression materials, condensation silicon impressions in the present study were made using stainless steel dies and putty/wash technique without tray adhesives, which is similar to making a clinical impression with stock tray. Using a stock tray, impression shrinkage results in oversized dies, which is advantageous in compensating forwax pattern and casting alloy shrinkage.^9^ Thus, the oversized die could be helpful in full seating of a casting crown. On the other hand, following disinfection of impressions, dimensional stability may change as a result of potential impression expansion. Thus, it is critical to maintain the balance between impression shrinkage and expansion and to know how much the dimensional accuracy of the impression material might be affected by the disinfection process. Al-Omari et al^20^ reported that changes of impressions produced by certain disinfectants were compensated by the setting expansion of the stone used to make the casts. This means that, provided they occur in the right direction, the changes of impressions and casts can compensate for each other, producing stone casts that are dimensionally closer to the original object than the impressions.^16^ For this to happen, the impression material should expand during disinfection to a degree analogous to the expansion that the stone mix would have on setting.^21^ There are many reasons for the dimensional changes in dental impression materials. All the elastomers exhibit a light contraction during polymerization as a result of the volume reduction due to the cross link and alcohol evaporation. This is only true for the condensation silicones. The incomplete elastic recovery may also give rise to impression with different dimensions compared to the original.^1^

In Spidex® samples of the present study, differences between the disinfected and non-disinfected conditions were significant. However, in Rapid® samples, there were no significant differences in any dimensions except for interpreparation distance among the control and the two disinfectant groups. The different behavior of the two impression materials may be related to the presence of certain ingredients such as surfactants in ample quantity in Rapid®, which reacted well to the disinfection, showing slight insignificant changes. The fact that both of these impression materials are condensation silicones indicates that extrapolation across different brands within a single generic group is not wise. This finding is in agreement with the results of Martin et al.^18^

The dimensional changes in the present study which represent expansion of tested impression materials following disinfection are in accordance with the findings of Thouati et al.^25^ Such an expansion can offset the polymerization shrinkage and therefore improve the precision of the resulting cast. Previous research also confirms the presence of an improvement in the precision of impressions in condensation silicone immersed in the disinfectant.^24^

Both spray disinfectants resulted in dimensional changes in the condensation silicone impressions. The alterations varied according to the disinfectant employed because the vaporization of alcohol as a by-product of polymerization is inhibited. Considering the measurements, it was observed that iodophor disinfectant did not affect each impression material similarly. In the case of impressions with Rapid®, the iodophor spraying resulted in an increase in the interpreparation dimension, more than that caused by hypochlorite spraying and in non-disinfected stone casts. It may be due to the expansion of the impression after disinfection and reduction of diameter of stone dies. Both of disinfectant materials caused a statistically significant increase in diameter and height in Spidex® impressions. Others have reported a decrease in the diameter of improved stone dies when disinfecting impressions with immersion disinfectants.^35^ It seems that the polymerization shrinkage of the Spidex® impression material is not completely negated by the use of a spray disinfectant, as demonstrated by the increase in percent change in the measurements.

The largest dimensional changes that occurred during the disinfection process were 0.4% in the interpreparation distance of Rapid®-iodophor, the height of Spidex®-iodophor and the diameter of Spidex®-iodophor groups. This finding is in accordance with the study of Johansen & Stackhause^36^ that showed the condensation silicone shrunk 0.44%. Thouati et al^25^ observed that the elastomer immersion in 5.25% sodium hypochlorite solution for 30 minutes caused expansion of the impressions, which is also in agreement with the result of present study.

There are also studies that are not in accordance with the findings of the present study. Adabo et al^8^ investigated the effect of disinfecting methods on the dimensional stability of six elastomeric materials and concluded that although there were significant differences among the elastomers used, the interaction between the material and the treatment was not significant. Matyas et al^37^ also showed that there were no significant dimensional changes when condensation silicone impressions were sprayed or immersed in the iodophor and chlorine compounds. This different result may be related to the use of different brands within a single generic group.

In the present study, dimensional changes that occurred during the disinfection process ranged from 0.1% to 0.4%. According to ADA specification 19 criteria, elastomeric impression materials should not exhibit more than 0.5% dimensional change within the first 24 hours.^38^ Thus, it seems although changes in the mean dimension measurements resulting from spray disinfections were significant, they are not great enough to cause critical positional distortion of teeth when fixed partial denture restorations are made.

A limitation of this study was that the impressions were made using a simulated crown preparation made of stainless steel, and thus clinical conditions could not be simulated. Under the simulated condition of the present study, unlike the natural oral environment, soft tissues, saliva and sulcular fluids were not present, and the temperature was also different from that of the oral cavity. Another limitation of this study was the use of sterilizable, metal full-arch impression stock trays, whereas, in some regions, disposable full-arch and dual-arch plastic trays are used.

## Conclusion

Based on the results of this study, the following conclusions were drawn:

1. There was a significant difference between the condensation silicone impression materials used. Rapid® has higher dimensional stability compared to that of the Spidex® after disinfection with two disinfectants.
2. Both of the impression samples showed significant difference in interpreparation distance.
3. Disinfection by iodophor resulted in more dimensional changes compared to sodium hypochlorite.
4. The dimensional changes of condensation silicone impression materials were lower than the maximum linear dimensional changes recommended by ISO 4823 (5%). Therefore, spray atomization disinfection technique with sodium hypochlorite and iodophor can be recommended for preservation of dimensional stability of the impression.
